# Validation of the Arabic version of the Dusseldorf Orthorexia Scale (DOS) among Lebanese adolescents

**DOI:** 10.1186/s40337-021-00488-4

**Published:** 2021-10-16

**Authors:** Radoslaw Rogoza, Souheil Hallit, Michel Soufia, Friederike Barthels, Sahar Obeid

**Affiliations:** 1 Cardinal Stefan Wyszyński University in Warsaw, Warsaw, Poland; 2 Research Department, Psychiatric Hospital of the Cross, Jal Eddib, Lebanon; 3 Faculty of Medicine and Medical Sciences, Holy Spirit University of Kaslik (USEK), Jounieh, Lebanon; 4 Institute of Experimental Psychology, Department of Clinical Psychology, Heinrich Heine University Düsseldorf, Düsseldorf, Germany; 5 Faculty of Arts and Sciences, Holy Spirit University of Kaslik (USEK), Jounieh, Lebanon

**Keywords:** Orthorexia nervosa, Adolescents, Düsseldorf Orthorexia scale, Arabic, Validation, Lebanon

## Abstract

**Background:**

Orthorexia Nervosa (ON) is defined as a pathological preoccupation characterized by obsessive beliefs and compulsive behaviors regarding 'pure' eating behaviors. Many scales have been established and display good results regarding reliability and validity measures, including but not limited to ORTO-R (revised version of ORTO-15), Eating Habits Questionnaire, Teruel Orthorexia Scale (TOS) and the Düsseldorf Orthorexia Scale (DOS). Among these, the DOS seems to be a promising measure for multiple reasons. The current paper aims to validate the DOS, a measure of ON, in a non-Western population of Lebanese adolescents.

**Methods:**

This was a cross-sectional designed study, conducted between May and June 2020, which enrolled 555 adolescents (15–18 years old; 75.7% females). Due the coronavirus pandemic outbreak, the data were gathered through snowball sampling using an online questionnaire. The DOS, TOS and ORTO-R scales were used in this study to screen for orthorexic tendencies and behaviors.

**Results:**

We tested four competing structural models of the DOS assessing its factorial validity. The results of the current investigation revealed that the one-factorial model is the best one to represent the structure of the questionnaire. We provided evidence for validity for the DOS through demonstrating that it correlates significantly with other measures of orthorexic behaviours (Teruel Orthorexia Scale and ORTO-R). Finally, we have gathered evidence that the orthorexic behaviours as measured by DOS are not associated with age (*r* = −.02; *p* = .589), household crowding index (*r* = .02; *p* = .578), and Body Mass Index (*r* = .04; *p* = .297). Yet, females as compared to males achieved higher scores (*M* = 20.07, *SD* = 6.38 vs *M* = 18.29, *SD* = 6.37; *p* = .005; *d* = .28).

**Conclusion:**

The Arabic version of the DOS seems to be a structurally valid and internally consistent questionnaire measuring orthorexic eating behavior in a sample of Lebanese adolescents. This tool may be useful for psychologists, psychiatrists, dietitians and other clinicians in the assessment and the treatment of the multidimensional nature of ON.

## Background

Orthorexia Nervosa (ON), which was originally proposed by Bratman in 1997 [1], is defined as a pathological preoccupation characterized by obsessive beliefs and compulsive behaviors regarding 'pure' eating behaviors [2]. The eventual aim in ON is to get a sensation of purity or well-being, without losing weight [3]. With time, people with ON spend longer time buying, scheduling, and preparing healthy meals, which in the long run turns into an obsession interfering with multiple aspects of life [4]. So far, ON is not acknowledged as a disorder in the Diagnostic and Statistical Manual of Mental Disorders, Fifth Edition (DSM-5) [5] or in the International Classification of Diseases, Eleventh Revision (ICD-11) [6]; however, a significant number of patients seek support from healthcare specialists for ON-related impairments [7–10]. Four criteria for ON have been suggested [11–15] and are summarized as follows: (a) obsession and concern for healthy eating, which consists in following a restrictive and 'pure' eating regimen; (b) excessive emotional distress accompanied by emotional state of guilt, shame and/or concern once the restrictive dietary rules followed by the subject have been violated; (c) physical impairments, more particularly nutritious scarcities may result in noteworthy weight loss, malnourishment and/or physical health complications; and (d) psychosocial impairments in social, professional and/or educational functioning.

The vast majority of research studies have assessed ON with the ORTO-15 [16]. However, the validity and reliability of this tool has been frequently questioned as for instance, it has an unstable factorial structure and is not suitable for the assessment of the prevalence of orthorexic behaviours [17, 18]. To address these issues, a revised version, that is the ORTO-R [19] was proposed, which although is not solving its parent measures limitations, it apparently reduces their influence [20]. In response to such difficulties, other scales such as the Eating Habits Questionnaire (EHQ) [21], the Düsseldorf Orthorexia Scale (DOS) [22], and the Teruel Orthorexia Scale (TOS) [23, 24] have been established and display good results regarding reliability and validity measures [23]. Among these, the DOS seems to be a promising measure for several reasons: (1) this tool is very short, hence, it is a time-efficient screening tool, (2) its items are short, easy and comprehensible, thus, it is suitable for individuals with lower education or for younger adolescents, (3) it has been validated in several languages, allowing comparisons between different countries and cultures, (4) it was created in a thorough and comprehensive process, starting from an item pool comprising almost 200 statements (the best items were gradually extracted in several studies and factor analyses). Some of the other questionnaires, on the other hand, are based on a smaller pre-selection or were constructed on the basis of expert opinions, which may have disadvantages.

Nevertheless, to these strengths of the DOS, there is also a one vital weakness, which we aim to address in the current study, namely its factorial validity. The one-dimensional structure of the DOS could only be partially confirmed in the original publication [22]. Subsequent studies provided ambiguous results. Some studies suggest that all items represent orthorexic behaviours, and as result, the one-factorial model describes the structure best [25]. Such model was endorsed even in light of the fit indices not being ideal [26]. Nevertheless, one might also find different propositions suggesting to retain two, three, or even five factors [27–29]. What is speaking against these models, however, is the fact that despite demonstrating multifactor structure and using oblique rotation methods, correlations between the latent factors were not reported in neither of the mentioned studies. Furthermore, while two different studies supported the three-factor model in two different populations [27, 28], in these studies, different items were loading on different factors, suggesting that the reported results are not driven by the theory, but rather are data-based. Finally, a model comprising five factors [26] hypothesized to reflect a structure of a ten-item measure seems to be overly complicated and ignoring the clinical construct features of the ON. Summing up, the one-factorial model of the DOS seems to be not only most theoretically and empirically convincing, but it also appears to be the most parsimonious solution. Nevertheless, to its potential advantages, to date, the DOS has been validated primarily in Western, educated, industrialized, rich, and democratic (i.e., WEIRD) populations.

To be noted that several socio-demographic factors have been correlated with ON [30, 31]. Regarding gender, ON symptomatology was significantly greater in women than men [32], even though one study has shown alike levels in females and males [33]. Regarding age, the results are controversial, with studies displaying either small or no significant negative associations [30, 34, 35]. The same is true for the effect of socio-economic status (SES), with studies showing a positive, negative or non-existent relationship between objective SES measures and ON [36–39]. Concerning Body Mass Index (BMI), a higher risk for ON is linked to both overweight and underweight [31].

### Current study

Our primary objective was to test the factorial structure, to assess the internal consistency and the convergent validity of the DOS among a sample of Lebanese adolescents. Noting that the DOS has been validated among US adolescents so far [40], revealing good face validity, and stating that the adjustment of two questions may be able to improve its face validity. The secondary objective was to identify sociodemographic factors that would be associated with ON in our sample. According to the literature, there are at least four different propositions of the DOS structure (i.e., comprising one-, two-, three-, and five factors). While some difficulties in achieving satisfactory model fit of the one-factor model are expected (e.g., Chard et al., 2019 [26]), we hypothesize that this particular model is the most adequate to represent the factorial structure (H1). While we acknowledge that models comprising more factors might potentially fit the data better, given theoretical (i.e., we operationalize orthorexic behaviors as an internally consistent construct), empirical (i.e., too high correlations between latent factors suggesting unity), and even practical (i.e., too few items per factor) arguments, we still prefer the one-factorial solution. We did not analyze a five-factor model, as it obviously ignores the clinical construct features of the ON. We also hypothesize (H2) that the orthorexic behaviours as captured by DOS, would be positively correlated with other measures of such (Teruel Orthorexia Scale which has good results regarding reliability and validity measures [23]; and items from ORTO-15, which were included in the ORTO-R to overcome at least some of the main limitations of the ORTO-15 [41]), providing support for its validity. In respect to socio-demographic variables, according to the literature review, we did not expect that scores would differ on the basis of age, household crowding index and BMI, however, based on the prior literature we did expect to observe that females would score higher on the DOS than males (H3).

## Methods

### Minimal sample size calculation

According to Comree and Lee [42], a minimum of 10 participants is needed for each scale’s item; since the DOS scale is composed of 10 items, a minimal number of 100 was needed for the factor analysis.

### Participants and procedure

This was a cross-sectional designed study, conducted between May and June 2020, which enrolled 555 adolescents residing in Lebanon (15 to 18 years old). The sample was distributed proportionate among all Lebanese governorates (Beirut, Mount Lebanon, North, South, and Bekaa). Due the coronavirus pandemic outbreak, the data were gathered through snowball sampling using an online questionnaire. The link was sent to adolescents from public and private schools. Prior to participation, study objectives and general instructions were delivered online for the individual subjects. No credits were received for participation.

The mean age of the participants was 16.66 ± 1.01 years, with 75.7% females. The mean house crowding index was 0.97 ± 0.51. In addition, the mean BMI was 22.33 ± 4.10; 77.4% of the adolescents had normal BMI, 17.4% were overweight, and 5.2% were obese. More details about the students can be found in Table 1.

**Table 1:** Sociodemographic and other characteristics of the participants (N = 555)

Variable	N (%)
*Gender*	
Male	135 (24.3%)
Female	420 (75.7%)
*District*	
Beirut	70 (12.6%)
Mount Lebanon	355 (64.0%)
North	84 (15.1%)
South	16 (2.9%)
Bekaa	30 (5.4%)

	Mean ± SD
Age (in years)	16.67 ± 1.00
Household crowding index	0.95 ± 0.50

The questionnaire was divided in three parts. In the first part, a written consent, confirming the approval of the adolescents and their parents to fill in the questionnaire was gathered. In the second part, respondents answered to questions assessing socio-demographic details (age, residency governorate, height, weight, etc.) and BMI. The Household Crowding Index (HCI), reflecting the socioeconomic status of the family, was calculated by dividing the number of persons living in the house by the number of rooms in the house; a higher HCI reflect a lower SES [43]. In the last part of the study, participants completed a set of self-report measures of ON.

## Measures

### Düsseldorf Orthorexia Scale (DOS)

The questionnaire was conceived in Arabic (Appendix 1), following the standard forward and back translation procedure (process involving two independent translations, synthesis of the two translations, back translations, and review of the pre-final version). The DOS comprises ten items, to which respondents answer on a four-point Likert scale where 1 = never, 2 = rarely, 3 = often, and 4 = always [44]. In this study, the internal consistency of the measure was good (α = 0.85).

### Teruel Orthorexia scale

The Teruel Orthorexia Scale (TOS), validated in Lebanon [45, 46], is a 17-item instrument that assesses ON with two separate dimensions [23]: 9 items for Healthy Orthorexia or “HeOr” (e.g., “I mainly eat foods that I consider healthy”) and 8 items for Orthorexia Nervosa or “OrNe” (e.g., “Thoughts about healthy eating do not let me concentrate on other tasks”). Responses are provided on a four-point Likert-type scale ranging from 0 = strongly disagree to 3 = strongly agree. Scores by dimension were computed as the sum of the item responses. In this study, the internal consistencies were 0.85 for the TOS OrNe and 0.83 for the TOS HeOr.

### ORTO

The ORTO questionnaire [16], which has been previously validated in Lebanon [47], is composed of 15 items, to which respondents answer using a four-point Likert scale 4-point Likert-scale ranging from 1 = always and 4 = never. Within the current study, we used the six items of the original scale (ORTO-R scale), which demonstrated best psychometric qualities [18, 19] and is validated in Lebanon [48]. In this study, the internal consistency of the measure was acceptable (α = 0.72).

### Statistical analyses

Since the data were collected using an online questionnaire, there were no missing values since responding to all questions was required. To assess the factorial validity, we used the confirmatory factor analysis (CFA) based on a polychoric correlation matrix (used to reflect the categorical character of the data). We used weighted least squares with means and variances adjusted (i.e., WLSMV) estimation. The comparative fit index (CFI) and the root mean square error of approximation (RMSEA) were used to evaluate the goodness-of-fit of the model [32]. RMSEA values ≤ 0.08 or CFI values > 0.90 indicate a good-fitting model. We estimated a total of four measurement models: 1) one-factorial model with all items entered as indicators of a single factor regarding orthorexic behaviours; 2) two correlated factors model [25], two competing three correlated factors models, accordingly to the results from Polish [27] and Chinese [28] populations. Full model specification, estimates of factor loadings, and factor correlations, are available as supplementary materials at the OSF project site (https://osf.io/ye9wz/?view_only=3bc73ffaa78e4d6cac4ce54e491fe0b3). Prior to the analyses, normality of distribution of the DOS score was confirmed via a calculation of the skewness and kurtosis. Values between − 2 and + 2 are considered acceptable to prove normal univariate distribution [49]. These conditions consolidate the assumptions of normality in samples larger than 300 [50]. In order to investigate the convergent validity of the DOS, Pearson’s correlations were calculated. The Student’s t-test was used to compare the two means.

## Results

The mean scores of the scales used in this study were as follows: DOS (19.64 ± 6.42), TOS orthorexia nervosa (5.23 ± 4.82), TOS healthy orthorexia (10.15 ± 5.83) and ORTO (15.16 ± 3.98).

### Factorial validity

The fit indices of the four tested CFA models are presented in Table 2. As can be seen, the one-factorial model fitted well according to CFI, but poorly according to RMSEA. Similar estimates were obtained for Models 2 and 3. Model 4 was the first that fitted well according to both statistics. However, the correlation between the latent factors almost equaled unity, questioning the sense of differentiation of such factors. Instead of suggesting a multidimensional structure, it rather suggests some sources of method bias. In fact, one pair of residuals (i.e., item 6 and 10) appeared to have a very large modification index (MI = 140.19). Moreover, this pair of items was an indicator of the same factor in all analyzed models, and even was the sole indicator of a factor in the best-fitting model (i.e., Model 4). Including such parameter in the one-factor model significantly improved the model fit (χ^2^_(34)_ = 163.39; *p* < 0.001; CFI = 0.970; RMSEA = 0.079 [0.067, 0.091]). Thus, although the one-factor model does not represent the best fit across all analyzed models, it apparently is the best model to describe the underlying structure of DOS, confirming our first hypothesis (H1).

**Table 2:** Fit indices of the four tested confirmatory factor analysis models of the Dusseldorf Orthorexia Scale (DOS)

	χ^2^_(df)_	*p*	CFI	RMSEA	90%CI	Factor correlation range	χ^2^_(df)_ difference test as compared to Model 1
Model 1	298.85_(35)_	.001	.939	.111	.099, .123	NA	–
Model 2	259.15_(34)_	.001	.948	.104	.092, .116	.87	39.70_(1)_***
Model 3	179.64_(32)_	.001	.966	.087	.075, .099	.77–.95	119.21_(3)_***
Model 4	138.02_(32)_	.001	.975	.073	.061, .086	.72–.95	160.83_(3)_***

Model 1 = one-factor; Model 2 = two correlated factors; Model 3 = three correlated factors [27]; Model 4 = three correlated factors [28]

***p < .001

### Convergent validity

Expectedly, as presented in Table 3, orthorexic behaviours as measured by the DOS were significantly correlated to other measures in a theoretically expected manner. Thus, the H2 was supported in full.

**Table 3:** Correlation of the DOS scale with other orthorexia scales

	Pearson correlation coefficient	*p*
TOS OrNe	0.715	< 0.001
TOS HeOr	0.754	< 0.001
ORTO	− 0.383	< 0.001

DOS = Düsseldorf Orthorexia Scale; TOS = Teruel Orthorexia Scale; The ORTO scores are reversed (lower scores indicate more orthorexia nervosa); negative correlation supports our hypothesis in this case

### Correlation of each item of the DOS scale with the total score

Each item of the DOS scale correlated well with the total score, with correlation coefficients varying between 0.463 and 0.769 (Table 4).

**Table 4:** Correlation of each item of the DOS scale with the total score

Variable	Item number	Item-total correlation
I feel upset after eating unhealthy foods	10	0.738
If I eat something, I consider unhealthy, I feel really bad	6	0.731
I find it difficult to go against my personal dietary rules	9	0.614
I have the feeling of being excluded by my friends and colleagues due to my strict nutrition rules	7	0.463
My thoughts constantly revolve around healthy nutrition and I organize my day around it	8	0.769
I have certain nutrition rules that I adhere to	2	0.673
I can only enjoy eating foods considered healthy	3	0.561
Eating healthy food is more important to me than indulgence/enjoying the food	1	0.644
I like that I pay more attention to healthy nutrition than other people	5	0.723
I try to avoid getting invited over to friends for dinner if I know that they do not pay attention to healthy nutrition	4	0.540

### Bivariate analysis of sociodemographic variables

The hypotheses for the associations between DOS scores and sociodemographic variables were not verified except for gender; higher DOS scores were significantly found in females compared to males (*M* = 20.07, *SD* = 6.38 vs *M* = 18.29, *SD* = 6.37; *p* = 0.005; Cohen’s *d* = 0.28), supporting the H3. DOS total score was not significantly associated with age (*r* = −0.02; *p* = 0.589), household crowding index (*r* = 0.02; *p* = 0.578), nor with BMI (*r* = 0.04; *p* = 0.297).

## Discussion

### Factorial validity: finding support for the one-factor model

Within this current study we scrutinized the psychometric properties of the DOS in a non-WEIRD Arabic population. Within the literature, it was unclear what the factorial structure of the measure is, with each study suggesting a different solution. We analyzed all of these models, highlighting their strengths but also illuminating their weaknesses. As a result, the current study serves as a guideline for future works, attempting to scrutinize the factorial structure of the DOS. Within this study, four competing measurement models present within the literature were tested [22–25]. Our findings revealed that although more complex models involving more factors are generally better fitted to the data, the more parsimonious one-factorial solution appears as preferable. The latent correlations between the factors were as high (i.e., 0.95) as unity, therefore, there is limited practical utility of differentiating these factors. Improvement in model fit, therefore, rather suggests some sources of method bias. We have successfully identified such source of method bias as the residuals of two items appeared to be highly correlated. This covariance was visible in previous research to the extent, that these two items were hypothesized to reflect a single factor [24]. Inclusion of such covariance term in the model resulted in a satisfactory fit of the one factor model. Thus, although within the literature different models of DOS appeared, we have provided evidence that the one-factorial model is the one that should be considered with regard to the DOS, confirming our first hypothesis.

### Internal consistency and convergent validity: further support for the model

This solution also appeared to be internally consistent, reaching a value of 0.85, which could be seen as satisfying and in line with the reliability reported for other translations [26–28, 51, 52] as well as for the original German version of the DOS [53]. Item-total correlations suggest that each item represents the DOS scale quite well. Regarding the validity of the proposed factorial solution, the DOS significantly correlated with the OrNe subscale of the TOS, which is supposed to assess orthorexic eating behavior, supporting the convergent validity of the DOS. However, to approximately the same extent, the DOS also correlated with the subscale HeOr, which is supposed to assess the non-pathological aspect of healthy eating [54]. To the best of our knowledge, there are no studies that have investigated the correlation between DOS and the TOS yet, hence, conclusions must be drawn with caution. This result could either suggest that the DOS also assesses the non-pathological aspect of healthy eating, which would impair its overall validity. On the other hand, since the two subscales HeOr and OrNe also intercorrelate to a high extent [54], the correlation of both subscales with the DOS could also indicate an unclear demarcation of the two constructs of “orthorexia nervosa” and “healthy orthorexia”. More research is needed to further investigate this aspect. Additionally, only a small correlation with the ORTO was observed [55, 56]. While we used the items included in a revised version of this scale, we used the original wording of the items and not the revised, which might have influenced the obtained results [48, 49]. Summing up, results obtained in the current study supports the validity of the DOS.

### Relations to socio-demographic variables: only gender is related to orthorexic behaviours

Regarding sociodemographic variables, the Arabic version of the DOS was not correlated with age, which is in line with the results of the original version of the DOS as well with translated versions [51, 53, 57, 58]. Furthermore, no correlation of the DOS with BMI could be observed, which also corresponds to studies investigating this relationship [26, 28, 51]. Regarding gender differences, the results of this study suggest that females display higher levels of orthorexic eating behavior than males. This result is in line with some previous studies [51, 53], but there are also studies revealing no difference between females and males [26] and in the Chinese version of the DOS, males displayed higher levels of orthorexic eating behavior [28]. Apart from sociocultural differences, which might explain the diverging results, there is a need for an in-depth investigation of possible gender differences because also regarding other questionnaires measuring orthorexic eating behavior, some studies did find a difference and some did not [for a review see 33], with a slight tendency towards females being more likely to display orthorexic eating behavior. Finally, no correlation of orthorexic eating behavior with the household crowding index could have been observed, which could be interpreted as an absence of a relation of orthorexic eating behavior to the socioeconomic status. Since this aspect has not been investigated with the DOS yet, there are no studies that we could compare our result to.

## Limitations

Since predominantly females participated in this study, the reported results are not generalizable to the whole adolescent Lebanese population. However, since eating disorders are more prevalent in females [59], the most relevant group in terms of the possible development of eating disordered has been captured. Nonetheless, future studies should aim at validating the Arabic version of the DOS in a more representative sample. Furthermore, the Arabic version of the DOS should also be validated in adult samples because the obtained results cannot be generalized to older populations. Because of the use of self-report questionnaires, an information bias might have occurred during data collection; the differences in the results might also be due to gender [60] and cultural [61] differences regarding attitudes towards eating. Future studies should also consider diagnostic interviews in order to assess orthorexic eating behavior and use the herewith obtained diagnosis for further validation purposes. The snowball sampling predisposes us to a selection bias. Moreover, specificity and sensitivity were not tested for, which makes it difficult to determine face validity. Future studies taking these limitations into consideration are warranted.

## Conclusions

The results of this study suggest that the DOS is a reliable and for the most part also a valid questionnaire to measure orthorexic eating behaviors. The one-factorial structure of the DOS is the most appropriate measurement model of the questionnaire and all future adaptations should consider it at the first place. Future research might consider modification of item content of the two correlated items identified in the study. This tool may be useful for psychologists, psychiatrists, dietitians and other clinicians in the assessment and the treatment of the multidimensional nature of ON.

## Data Availability

All data generated or analyzed during this study are not publicly available to maintain the privacy of the individuals’ identities. The dataset supporting the conclusions is available upon request to the corresponding author.

## Abbreviations

DOS: Düsseldorf Orthorexia Scale
ON: Orthorexia Nervosa
ICD-11: International Classification of Diseases, Eleventh Revision
EHQ: Eating Habits Questionnaire
SES: Socio-economic status
BMI: Body Mass Index
HCI: Household Crowding Index
TOS: Teruel Orthorexia Scale
TOS HeOr: TOS Healthy Orthorexia
TOS OrNe: TOS Orthorexia Nervosa
CFA: Confirmatory factor analysis
WLSMV: Weighted least squares with means and variances adjusted
CFI: Comparative fit index
RMSEA: Root mean square error of approximation

## References

[1] Bratman S (1997). Health food junkie. Yoga J.

[2] Bratman S, Knight D (2000). Health food junkies: overcoming the obsession with healthful eating.

[3] Brytek-Matera A (2012). Orthorexia nervosa–an eating disorder, obsessive-compulsive disorder or disturbed eating habit. Arch Psychiatry Psychother.

[4] Oberle CD, Samaghabadi RO, Hughes EM (2017). Orthorexia nervosa: assessment and correlates with gender, BMI, and personality. Appetite.

[5] American Psychiatric Association (2013). Diagnostic and statistical manual of mental disorders.

[6] World Health Organization (2018) International classifcation of diseases for mortality and morbidity statistics (11th rev). Available https://icd.who.int/browse11/l-m/en. Accessed 1 Mar 2020.

[7] Vandereycken W (2011). Media hype, diagnostic fad or genuine disorder? Professionals' opinions about night eating syndrome, orthorexia, muscle dysmorphia, and emetophobia. Eat Disord.

[8] Reynolds R, McMahon S (2019). Views of health professionals on the clinical recognition of orthorexia nervosa: a pilot study. Eat Weight Disorders-Stud Anorexia, Bulimia Obesity.

[9] Ryman FVM, Cesuroglu T, Bood ZM, Syurina EV (2019). Orthorexia Nervosa: disorder or not? Opinions of Dutch Health Professionals. Front Psychol.

[10] Barthels F, Lavendel S, Müller R, Pietrowsky R (2019). Relevance of orthorexic eating behavior in nutrition conseling and nutrition therapy. Results of a nationwide survey among German nutrition-ists. Ernahrungs Umschau.

[11] Dunn TM, Bratman S (2016). On orthorexia nervosa: a review of the literature and proposed diagnostic criteria. Eat Behav.

[12] Moroze RM, Dunn TM, Holland C, Yager J, Weintraub P (2014). Microthinking about micronutrients: a case of transition from obsessions about healthy eating to near-fatal" orthorexia nervosa" and proposed diagnostic criteria. Psychosomatics.

[13] Barthels F, Meyer F, Pietrowsky R (2015). Duesseldorf orthorexia scale–construction and evaluation of a questionnaire measuring orthorexic eating behavior. Z Klin Psychol Psychother.

[14] Setnick J (2013). The Eating Disorders Clinical Pocket Guide, Second Edition: Quick Reference for Healthcare Providers.

[15] Obeid S, Hallit S, Akel M, Brytek-Matera A (2021). Orthorexia nervosa and its association with alexithymia, emotion dysregulation and disordered eating attitudes among Lebanese adults. Eat Weight Disord.

[16] Donini LM, Marsili D, Graziani MP, Imbriale M, Cannella C (2005). Orthorexia nervosa: validation of a diagnosis questionnaire. Eat Weight Disord.

[17] Roncero M, Barrada JR, Perpina C (2017). Measuring orthorexia nervosa: psychometric limitations of the ORTO-15. Span J Psychol.

[18] Rogoza R (2019). Investigating the structure of ORTO-15: a meta-analytical simulation study. Eat Weight Disord.

[19] Rogoza R, Donini LM (2020). Introducing ORTO-R: a revision of ORTO-15: Based on the re-assessment of original data. Eat Weight Disord.

[20] Donini LM, Marsili D, Graziani MP, Imbriale M, Cannella C (2004). Orthorexia nervosa: a preliminary study with a proposal for diagnosis and an attempt to measure the dimension of the phenomenon. Eat Weight Disord.

[21] Gleaves DH, Graham EC, Ambwani S. Measuring “orthorexia”: development of the eating habits questionnaire. Int J Educ Psychol Assess. 2013.

[22] Barthels F, Meyer F, Pietrowsky R (2015). Orthorexic eating behavior. A new type of disordered eating. Ernahrungs Umschau.

[23] Barrada JR, Roncero M (2018). Bidimensional structure of the orthorexia: development and initial validation of a new instrument. Anales De Psicología/Ann Psychol.

[24] Depa J, Barrada JR, Roncero M (2019). Are the motives for food choices different in orthorexia nervosa and healthy orthorexia?. Nutrients.

[25] Meule A, Holzapfel C, Brandl B, Greetfeld M, Hessler-Kaufmann JB, Skurk T, Quadflieg N, Schlegl S, Hauner H, Voderholzer U (2020). Measuring orthorexia nervosa: A comparison of four self-report questionnaires. Appetite.

[26] Chard CA, Hilzendegen C, Barthels F, Stroebele-Benschop N (2019). Psychometric evaluation of the English version of the Dusseldorf Orthorexie Scale (DOS) and the prevalence of orthorexia nervosa among a U.S. student sample. Eat Weight Disord.

[27] Brytek-Matera A (2020). The Polish version of the Dusseldorf Orthorexia Scale (PL-DOS) and its comparison with the English version of the DOS (E-DOS). Eat Weight Disord.

[28] He J, Ma H, Barthels F, Fan X (2019). Psychometric properties of the Chinese version of the Dusseldorf Orthorexia Scale: prevalence and demographic correlates of orthorexia nervosa among Chinese university students. Eat Weight Disord.

[29] Brytek-Matera A, Sacre H, Staniszewska A, Hallit S (2020). The Prevalence of Orthorexia Nervosa in Polish and Lebanese adults and its relationship with sociodemographic variables and BMI ranges: a cross-cultural perspective. Nutrients.

[30] McComb SE, Mills JS (2019). Orthorexia nervosa: a review of psychosocial risk factors. Appetite.

[31] Strahler J, Stark R (2019). Orthorexia nervosa: Verhaltensauffälligkeit oder neue Störungskategorie?. Suchttherapie.

[32] Haddad C, Obeid S, Akel M, Honein K, Akiki M, Azar J, Hallit S (2019). Correlates of orthorexia nervosa among a representative sample of the Lebanese population. Eat Weight Disord.

[33] Strahler J (2019). Sex differences in orthorexic eating behaviors: A systematic review and meta-analytical integration. Nutrition.

[34] Brytek-Matera A, Staniszewska A, Hallit S (2020). Identifying the profile of orthorexic behavior and "normal" eating behavior with cluster analysis: a cross-sectional study among Polish adults. Nutrients.

[35] Awad E, Salameh P, Sacre H, Malaeb D, Hallit S, Obeid S (2021). Association between impulsivity and healthy orthorexia: any moderating role of personality traits?. Psychol Health Med.

[36] Missbach B, Hinterbuchinger B, Dreiseitl V, Zellhofer S, Kurz C, Konig J (2015). When eating right, is measured wrong! A validation and critical examination of the ORTO-15 questionnaire in German. PLoS ONE.

[37] Dell'Osso L, Abelli M, Carpita B, Massimetti G, Pini S, Rivetti L, Gorrasi F, Tognetti R, Ricca V, Carmassi C (2016). Orthorexia nervosa in a sample of Italian university population. Riv Psichiatr.

[38] Dittfeld A, Gwizdek K, Jagielski P, Brzek J, Ziora K (2017). A Study on the relationship between orthorexia and vegetarianism using the BOT (Bratman Test for Orthorexia). Psychiatr Pol.

[39] Strahler J, Haddad C, Salameh P, Sacre H, Obeid S, Hallit S (2020). Cross-cultural differences in orthorexic eating behaviors: associations with personality traits. Nutrition.

[40] King E, Wengreen H, Litchford A, Bailey C, Beck C, Yan P, Edwards M, Hudson R, Kunzler A, Matthias H (2020). Validating the Düsseldorf Orthorexie Scale (DOS) for use in adolescents aged 14–17. Curr Dev Nutr.

[41] Ozdengul F, Yargic MP, Solak R, Yaylali O, Kurklu GB (2021). Assessment of orthorexia nervosa via ORTO-R scores of Turkish recreational and competitive athletes and sedentary individuals: a cross-sectional questionnaire study. Eat Weight Disord.

[42] Comrey AL, Lee HB (2013). A first course in factor analysis.

[43] Melki IS, Beydoun HA, Khogali M, Tamim H, Yunis KA, Neonatal NCP (2004). Household crowding index: a correlate of socioeconomic status and inter-pregnancy spacing in an urban setting. J Epidemiol Community Health.

[44] Barthels F, Meyer F, Pietrowsky R. Die Düsseldorfer Orthorexie Skala–Konstruktion und Evaluation eines Fragebogens zur Erfassung ortho-rektischen Ernährungsverhaltens. Zeitschrift für Klinische Psychologie und Psychotherapie. 2015

[45] Awad E, Obeid S, Sacre H, Salameh P, Strahler J, Hallit S. Association between impulsivity and orthorexia nervosa: any moderating role of maladaptive personality traits? Eat Weight Disord. 2021 Apr 11. doi: 10.1007/s40519-021-01186-5 10.1007/s40519-021-01186-5 33840074

[46] Mhanna M, Azzi R, Hallit S, Obeid S, Soufia M (2021). Validation of the Arabic version of the Teruel Orthorexia Scale (TOS) among Lebanese adolescents. Eat Weight Disord.

[47] Haddad C, Hallit R, Akel M, Honein K, Akiki M, Kheir N, Obeid S, Hallit S (2020). Validation of the Arabic version of the ORTO-15 questionnaire in a sample of the Lebanese population. Eat Weight Disord.

[48] Hallit S, Brytek-Matera A, Obeid S (2021). Orthorexia nervosa and disordered eating attitudes among Lebanese adults: assessing psychometric proprieties of the ORTO-R in a population-based sample. PLoS ONE.

[49] George D (2011). SPSS for windows step by step: a simple study guide and reference, 17.0 update, 10/e.

[50] Mishra P, Pandey CM, Singh U, Gupta A, Sahu C, Keshri A (2019). Descriptive statistics and normality tests for statistical data. Ann Card Anaesth.

[51] Ferreira C, Coimbra M (2020). To further understand orthorexia nervosa: DOS validity for the Portuguese population and its relationship with psychological indicators, sex, BMI and dietary pattern. Eat Weight Disord.

[52] Parra-Fernandez ML, Onieva-Zafra MD, Fernandez-Munoz JJ, Fernandez-Martinez E (2019). Adaptation and validation of the Spanish version of the DOS questionnaire for the detection of orthorexic nervosa behavior. PLoS ONE.

[53] Barthels F, Meyer F, Pietrowsky R (2015). Die Düsseldorfer Orthorexie Skala - Konstruktion und Evaluation eines Fragebogens zur Erfassung orthorektischen Ernährungsverhaltens [Duesseldorf Orthorexia Scale – Construction and Evaluation of a Questionnaire Measuring Orthorexic Eating Behavior]. Z Klin Psychol Psychother.

[54] Barrada JR, Roncero M (2018). Bidimensional structure of the orthorexia: development and initial validation of a new instrument. Anales de Psicología/Ann Psychol.

[55] Donini LM, Marsili D, Graziani MP, Imbriale M, Cannella C (2004). Orthorexia nervosa: A preliminary study with a proposal for diagnosis and an attempt to measure the dimension of the phenomenon. Eat Weight Disord.

[56] Donini LM, Marsili D, Graziani MP, Imbriale M, Cannella C (2005). Orthorexia nervosa: validation of a diagnosis questionnaire. Eat Weight Disord.

[57] Parra-Fernández ML, Onieva-Zafra MD, Fernández-Muñoz JJ, Fernández-Martínez E (2019). Adaptation and validation of the Spanish version of the DOS questionnaire for the detection of orthorexic nervosa behavior. PLoS ONE.

[58] Brytek-Matera A (2020). The Polish version of the Düsseldorf Orthorexia Scale (PL-DOS) and its comparison with the English version of the DOS (E-DOS). Eat Weight Disord.

[59] Hoek HW, Van Hoeken D (2003). Review of the prevalence and incidence of eating disorders. Int J Eat Disorder.

[60] Zakhour M, Haddad C, Sacre H, Tarabay C, Zeidan RK, Akel M, Hallit R, Kheir N, Obeid S, Salameh P, Hallit S (2021). Differences in the associations between body dissatisfaction and eating outcomes by gender? A Lebanese population study. Rev Epidemiol Sante Publique.

[61] Swanson SA, Saito N, Borges G, Benjet C, Aguilar-Gaxiola S, Medina-Mora ME, Breslau J (2012). Change in binge eating and binge eating disorder associated with migration from Mexico to the U.S. J Psychiatr Res.

